# Influence of ultrasound, with and without water spray cooling, on removal of posts cemented with resin or glass ionomer cements: An *in-vitro* study

**DOI:** 10.4103/0972-0707.71641

**Published:** 2010

**Authors:** M S Adarsha, D A Lata

**Affiliations:** Department of Conservative Dentistry and Endodontics, V.S. Dental college and Hospital, Bangalore, India; 1 Department of Conservative Dentistry and Endodontics, M.R. Ambedkar Dental College and Hospital, Bangalore, India

**Keywords:** Cast metal posts, GIC, resin cement, traction force, ultrasound

## Abstract

**Aims/objectives:**

To evaluate *in vitro* the ultrasonic vibration efficacy with and without water spray cooling on the reduction of the amount of force necessary to dislodge the cast posts cemented with resin cement and to compare it with those cemented with GIC Type I luting cement.

**Materials and Methods:**

Sixty samples were divided into six groups: groups 1, 2, 3, posts cemented with GIC; groups 4, 5, 6, posts cemented with resin; groups 1 and 4 (controls), no ultrasound; groups 2 and 5, ultrasound without water spray; and groups 3 and 6, ultrasound with water spray. Instron testing machine was used to dislodge the posts from the root canals and the data was statistically analyzed.

**Results:**

Ultrasound with water spray (group 3) among the GIC groups reduced the traction force necessary to extract posts by 53.33% whereas ultrasound without water spray (group 5) among the resin groups reduced by 59.5% compared to control.

**Conclusion:**

Ultrasound with water is more effective in removing posts cemented with GIC because of the ultrasonic energy being transferred to the post. Ultasonics without water is more effective in removing posts fixed with resin cement probably by the indirect action of heat production.

## INTRODUCTION

When endodontic failure occurs, conservative orthograde retreatment is usually the preferred treatment choice rather than periapical surgery, since re-treatment is generally more successful and most predictable. Teeth with intraradicular posts in need of re-treatment continue to present a challenge to clinicians. Non-surgical retreatment, normally considered to be more conservative than periradicular surgery, can result in catastrophic consequences, such as weakening of the root, perforation, fracture of the remaining root structure or damaging the supporting tissues in an attempt to remove a well-cemented post. The degree of difficulty will vary according to the post type; custom or prefabricated, post design; parallel, tapered, smooth, serrated, or threaded, as well as post-length and cementing agent.[1]

Several techniques and instruments have been recommended to remove posts. Some examples of post removal devices are the Masserann Kit, the Eggler post remover, the Ganon post remover, which is also called the Thomas Extracteur De Pivots and more recently, the Ruddle post remover.[2] These methods and procedures, although effective in removing intraradicular posts, may occasionally fracture the root, remove too much dental structure, or perforate the root.

The emergence of ultrasonics and its implementation into endodontics have provided clinicians with a useful adjunct for post removal. Several authors have suggested the use of ultrasound alone or in combination with other techniques to make this procedure safer.[3–7]

All posts, irrespective of their retentive qualities, require a luting cement to seal the irregularities between the post and the canal walls. Currently, four main types of commercial cementing agents are used for posts: zinc phosphate cement, GIC, zinc polycarboxylate cement and resin cement.

Resin cements provide the highest retention for posts compared to other types of cement. Despite these desirable characteristics, clinicians who use resin cements must be aware of the difficulty in their removal from the root canal if necessary.

The use of heat as an auxiliary aid to remove posts fixed with resin cement has been suggested.[1] Water spray is a common technique to minimize heat generation during application of ultrasonic forces. However, there is a gap in knowledge about whether water spray and ultrasonic forces interact to alter the force required for removal of resin-cemented posts. Garrido *et al*,[8] have demonstrated the efficacy of ultrasonic vibrations for removing posts. They also showed that cooling affects ultrasound efficacy depending on the type of cement used to lute the metal posts.

Therefore, the aim of the present study is to evaluate *in vitro* the ultrasonic vibration efficacy with and without water spray cooling on the reduction of the amount of force necessary to dislodge the cast posts cemented with resin cement and to compare it with those cemented with Glass Ionomer Type I luting cement.

## MATERIALS AND METHODS

Sixty human maxillary canines with root lengths of at least 15mm measured from the apex to the CEJ, extracted because of periodontal problems, were selected and stored in distilled water. All teeth were free of caries and fractures. These teeth were sectioned transversally 2mm above the proximal CEJ using a diamond disc. The root canals were prepared to a working length 0.5mm short of apical foramen with K-type files up to size 50 diameter. During cleaning and shaping, 3% NaOCl and 17% EDTA (RC Prep) were used to irrigate the canal and facilitate instrumentation. The root canals were obturated with gutta-percha points and sealer AH 26 cement (Dentsply) using warm lateral compaction technique. After obturation, the canals were sealed with Cimpat Pink (Septodont), and samples were kept in distilled water at room temperature for 7 days. Subsequently, for post space preparation, the coronal 10 mm of each root canal was instrumented with size 1-5 peeso reamers to a diameter of 1.5 mm. The final dowel diameter was 1.5mm through out the length of the post space which corresponds to size 5 peeso reamer. A guideline was also used for crown restoration using a shoulder margin in the cervical portion, 2mm from the coronal surface and 1mm wide 60-degree bevel at the tooth-core junction was placed.

Direct post and core patterns were made with inlay wax. The post was 10mm long and the core was 5mm. Trapezoidal shaped attachments with a hole were provided at the end of the core on all specimens to adapt to the instron testing machine. Impressions were cast in Ni-Cr dental casting alloy (Argeloy-ARGEN)), castings cleaned with an air-abrasive containing 50 *μ*m aluminum oxide particles.

### Distribution of samples

Samples were divided randomly into six groups of ten teeth each. Posts from groups 1, 2, and 3 were fixed with GIC (Ketac cem, 3M), and posts from groups 4, 5, and 6 were fixed with resin cement (Rely X ARC, 3M). All samples were then stored in 100% humidity at room temperature for 3 weeks before receiving the following treatments:

Group 1: posts fixed with GIC (Ketac Cem), no ultrasound (control group).

Group 2: posts fixed with GIC (Ketac Cem), ultrasound without water spray.

Group 3: posts fixed with GIC (Ketac Cem), ultrasound with water spray.

Group 4: posts fixed with resin cement (Rely X ARC), no ultrasound (control group).

Group 5: posts fixed with resin cement (Rely X ARC), ultrasound without water spray.

Group 6: posts fixed with resin cement (Rely X ARC), ultrasound with water spray.

The specimens were mounted into an aluminum mould using self-cure acrylic resin and color coded. Dental surveyor was used in mounting the specimens to enable subsequent post removal in a direction parallel to the long axis of the post and cores.

Ultrasound was applied with EMS scaler unit using endodontic ultrasonic tip – ProUltra Endo tip No.1 (Dentsply). Samples were stabilized in a bench vise, and the ultrasonic unit was set to maximum power and applied for 15 sec at buccal, lingual, and proximal surfaces of the tooth core interface with a gap of 30 sec between each application. This procedure was repeated once more. Hence, the ultrasound was applied for a total duration of 30 sec on each surface intermittently, controlled with a timer. Each specimen received the vibrations for a total duration of 2 min.

The posts were subjected to increasing traction forces 1 mm/ min using Instron testing machine until their displacement from the root. Data was recorded in kiloNewtons and subsequently analyzed using ANOVA variance with post hoc test, Student “*t*” test and effect size Cohen “d” test.

## RESULTS

All the specimens were subjected to tensile force using an Instron universal testing machine and the load at which post and cores dislodged from the canals was recorded in KiloNewtons.

In the resin groups, the traction force required for the group of ultrasonics without water was less than that required for the group with water whereas in the GIC groups, the traction force required for the group of ultrasonics with water was less than that required for the group without water.

Groups in which posts were cemented with Rely X ARC resin cement, ultrasonic vibration without water spray (Group 5) reduced the traction force necessary to remove the posts by 59.5% of that required in the control group. But the comparison between the control and the ultrasonic with water (Group 6) showed 13.3% reduction.

On the contrary, among the groups cemented with Ketac Cem GIC, ultrasonic vibration with water spray (Group 3) reduced the traction force necessary to dislodge the post by 53.33% of that required in the control group. The comparison between the control and the ultrasonic without water (Group 2) showed 23.56% reduction.

## DISCUSSION

There have been many studies suggesting techniques and instruments to facilitate the removal of intraradicular posts. Despite the large number of techniques available, priority has been given to those that carry a lower risk of root fracture and perforation, involve less drilling of the remaining dental structure and that can be used for both anterior and posterior teeth. To overcome the difficulties involved in the removal of metal intraradicular posts and to make this procedure safer, ultrasonic vibration has been suggested as an alternative technique for removal, either alone or in combination with other techniques.[5,8,9] Ultrasound is a mechanical form of energy where the ultrasonic oscillations are generated in the device and transferred to the cast post, with the objective of “disrupting” the cement bond between the post and the walls of the canal. Once the cement bond is broken, removal of the post is facilitated.[3,7,10] Ultrasound is considered as the safest and most efficient technique because it saves time, there is minimal wearing of tooth structure, there is low risk of fracture or perforation and the technique is applicable to all teeth.[5,11]

**Figure 1:**
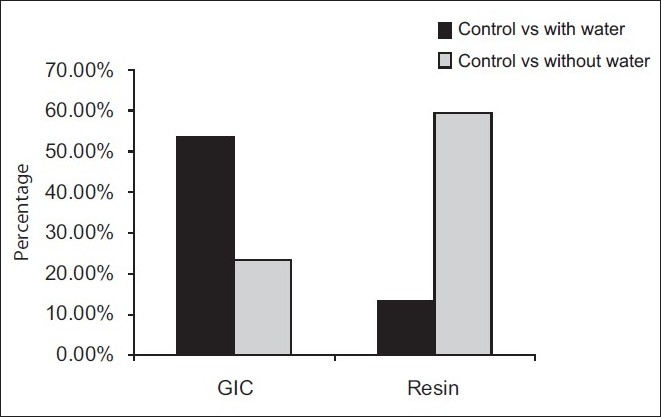
Percentage decrease in traction forces

Comparison of results of ultrasound (Groups 2 and 3) with those of group without ultrasound (Group 1) confirmed that the ultrasonic vibration reduces the force necessary to remove the cast metal post cemented with GIC independent of whether water cooling was used or not [Figure 1]. Groups in which the posts were cemented with GIC, ultrasound applied with water spray cooling, (Group 3) reduced the traction force necessary to extract posts by 53.33% when compared to control (Group 1). However, ultrasonic vibration without water spray (Group 2) resulted in 23.56% reduction in traction forces as against control [Figure 1].

These results are in conformity with those obtained by Gomes *et al*,[1] Buoncristini *et al*,[5] and Johnson *et al*,[10] who demonstrated the efficacy of the ultrasonic device in the removal of posts cemented with zinc phosphate cement and GIC cement.

Posts fixed with GIC were less resistant to traction forces following ultrasound with water because the mechanical impact of the ultrasound was transferred to the post, probably breaking down the surrounding cement, thus minimizing the force necessary to dislodge the posts.[3,10] Water spray must also be considered because it affects the solubility of GIC, thus promoting the solubility of the cement. The relative inefficiency of ultrasound without water spray on posts cemented with GIC is probably related to the low thermal expansion values for this luting cement.

**Figure 2:**
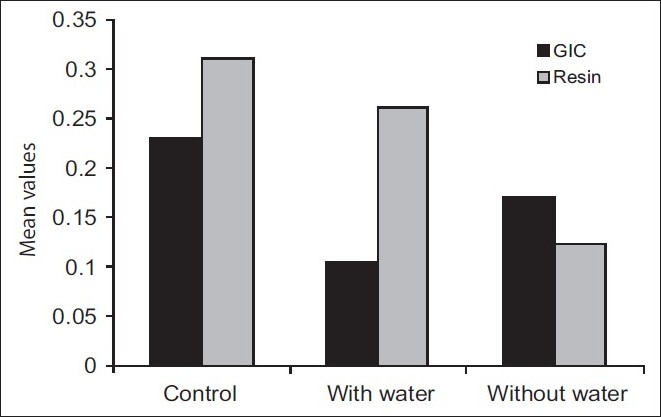
Means of traction forces in the control and test groups (kN)

Ultrasonic vibration with water spray (Group 6) did not present statistically significant difference (*P*>0.01) in posts fixed with resin cement when compared with the control group (Group 4), [Table 1 and Figure 2]. This is in agreement with the findings of Gomes *et al*,[1] and Chandler *et al*,[12] who found that the application of ultrasonic vibration did not influence the retention of posts cemented with resin cement. According to Phillips,[13] resin cements are not friable and do not produce micro fractures, as is seen with GIC and zinc phosphate.

**Table 1:** Means of traction forces in the different groups (kN)

Groups	GIC (Mean±SD)	Resin (Mean±SD)	*P* value (Student test)
Control (A)	0.225±0.03	0.301±0.05	<0.001**
With water (B)	0.105±0.03	0.261±0.02	<0.01**
Without water (C)	0.172±0.02	0.122±0.03	0.003**
Significance (ANOVA)	F=37.645; p<0.001**	F=63.106; p<0.001	-

The fact that ultrasonic vibrations without water spray (Group 5) resulted in statistically significant differences (*P*<0.001**) in posts fixed with resin cements when compared to the control group (Group 4) is in agreement with the findings of Garridio *et al*,[8] probably by indirect action of heat production, and not by its movement. Ultrasound without water spray (Group 5) among the resin groups reduced the traction forces by 59.5% compared to control (Group 4) [Figure 1]. Resin cements are susceptible to temperature changes because of their high thermal expansion property.

The heat generated by the ultrasonic tip and post metallic alloy is easily transmitted to the luting agents because of high thermal conductance of metals. Watanable *et al*,[14] observed that the adhesion capacity of a resin cement reduces gradually with the number of thermal cycles.

Resin cement retention was significantly larger than GIC [Figure 2]. These results were similar to those obtained by Gomes *et al*,[1] Chan *et al*,[15] and soares *et al*,[16] who reported that the resin cement; Panavia EX gave better results in relation to the retention of the cemented posts compared to Ketac-Cem, GIC cement.

Few *in vitro* studies have raised concerns over the use of ultrasound without water as it could lead to temperature rise enough to cause damage to the periodontal tissues.[17,18] In the present study, ultrasound was applied intermittently for 15 sec on each surface of the post and core with a gap of 30 sec between each application and the procedure was repeated once more.

Dominici *et al*,[17] showed that heat transmission through the post and dentine resulted in temperature increase of 9.5°C in 15 sec along the external root surface. Ultrasonic application to the post for longer than 15 sec generated higher temperature on the root surface. The threshold for heat induced bone necrosis is 10°C above normal body temperature of 37°C sustained for 1 min.[19] Hence, it was decided not to use ultrasound continuously for more than 15 sec to avoid temperature rise above 10°C.

However, it is not known whether 30 sec rest between 15 sec of ultrasonic application without water spray would produce periodontal injury or not because of heat. Further research in this area should be pursued. Further studies should also focus on determining differences in temperature rise comparing different teeth, varying dentine and enamel thickness, a range of clinical techniques including a range of applied force and different coolant conditions. The intensity of temperature rise, the frequency of the thermal insult, the size of the thermal mass and the duration of the temperature rise are all important factors to be considered in determining adverse thermal effects to tissue.

In a clinical setting, it could be expected that the temperature would be lower, given the larger body mass for heat dissipation and the thermoregulatory mechanism such as capillary blood flow and tissue metabolism. These factors are likely to have an impact in reducing the amount of heat that is actually absorbed by periodontal tissues.

The working hypothesis in this study is that either ultrasonics with or without water spray cooling does not alter the force required for removal of posts cemented with GIC and resin cement. However, this experimental premise was disproved and the presence or absence of water spray interferes in the efficacy of the ultrasound, depending on the type of cement used, and can reduce the force necessary to extract posts by approximately 59.5% increasing the predictability of success.

## CONCLUSION

Within the limitations of this study, it can be concluded that:

- Ultrasonic vibration using ProUltra endodontic tip no 1 reduced the force necessary to remove the cast metal posts cemented with GIC independent of whether water cooling was used or not.
- Ultrasound with water is more effective in removing posts cemented with GIC because of the ultrasonic energy being transferred to the post, probably breaking down the surrounding cement and the water spray promoting the solubility of the GIC, and
- Ultrasonics without water is more effective in removing posts fixed with resin cement probably by the indirect action of heat production compromising their chemical properties of adhesion and consequently, mechanical retention.

However, it is not known whether 30 sec rest between 15 sec of ultrasonic application without water spray would produce periodontal injury or not because of heat. Further research in this area should be pursued and the results of this study should be interpreted with caution when used in the clinical situation.
